# Dr. Fitch Brewer

**Published:** 1917-07

**Authors:** 


**Dr. Fitch Brewer,** Buffalo 1913, of Buffalo, died at the Sisters' Hospital, following an operation for appendicitis, June 8.

